# Supplementary material for: The Helicobacter pylori HspR-Modulator CbpA Is a Multifunctional Heat-Shock Protein
Source: Microorganisms. 2020 Feb 13;8(2):251. doi: 10.3390/microorganisms8020251 (PMC7074700; doi:10.3390/microorganisms8020251)
Supplement: Supplementary file 1 [file microorganisms-08-00251-s001.pdf]

Supplementary material

The *Helicobacter pylori* HspR-modulator CbpA is a multifunctional heat-shock protein

Simona Pepe, Vincenzo Scarlato* and Davide Roncarati*

Table of contents
Pag.

Figure S1: Analysis of CbpA and DnaJ *in vitro* ATPase activity…………………………………………….…….. 2

Figure S2: *In vitro* crosslinking assay of CbpA-wt and CbpA-ΔCt proteins with Dithiobis[succinimidylpropionate] (DSP) homobifunctional reagent……………………………………………. 3

Figure S1

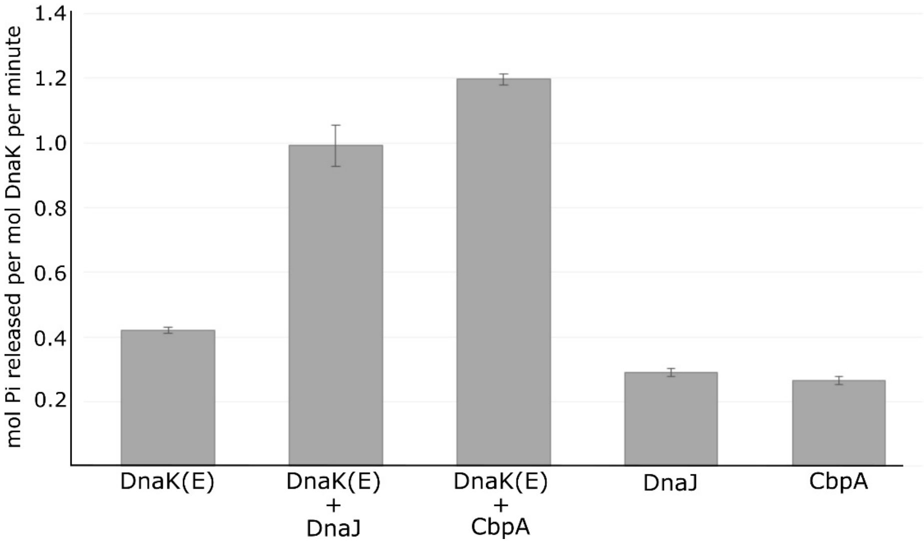

Figure S1. Analysis of CbpA and DnaJ *in vitro* ATPase activity. In order to exclude contamination of protein preparations by ATPases, CbpA and DnaJ purified proteins were analysed for their ATPase activities and the results are here reported together with ATPase activity measured for DnaK alone (DnaK (E)), with DnaJ (DnaK (E) + DnaJ) or with CbpA (DnaK (E) + CbpA). Standard error was calculated from three independent experiments. The (E) symbol indicates the presence of the nucleotide exchange factor GrpE.

Figure S2

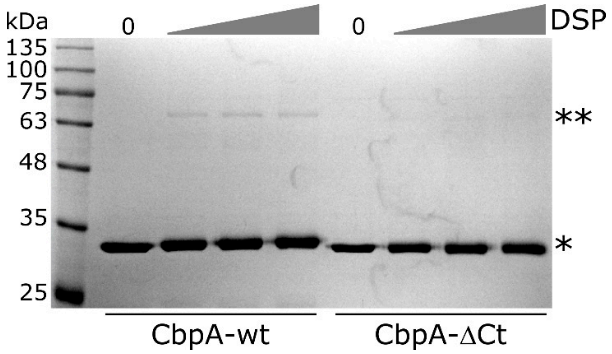

Figure S2. *In vitro* crosslinking assay of CbpA-wt and CbpA-ΔCt proteins with Dithiobis [succinimidylpropionate] (DSP) homobifunctional reagent. SDS-PAGE of the purified CbpA-wt and CbpA-ΔCt proteins not treated (lane 0) or treated with increasing concentrations of Dithiobis [SuccinimidylPropionate] (DSP) homobifunctional reagent (Pierce, Rockford, Illinois, USA). The purified CbpA-wt and CbpA-ΔCt proteins were diluted to 3.6 μM with 1XPBS and then incubated with increasing concentrations of DSP (0, 45, 90, 180 μM from left to right) for 30 minutes at 25°C. Chemical crosslinking was halted by adding 2 μl of 1M Tris-HCl pH 7.5. Then, reactions were incubated at 100°C for 5 minutes, separated by SDS-PAGE and stained with Coomassie Brilliant Blue R-250 (Sigma-Aldrich, St Louis, Missouri, USA). Symbols: *, band of the monomeric form of the proteins; **, band of the putative dimeric form of the CbpA-wt protein.
